# Synergistic Effect of *Beauveria bassiana* and *Trichoderma asperellum* to Induce Maize (*Zea mays* L.) Defense against the Asian Corn Borer, *Ostrinia furnacalis* (Lepidoptera, Crambidae) and Larval Immune Response

**DOI:** 10.3390/ijms21218215

**Published:** 2020-11-03

**Authors:** Raufa Batool, Muhammad Jawad Umer, Yangzhou Wang, Kanglai He, Tiantao Zhang, Shuxiong Bai, Yang Zhi, Jie Chen, Zhenying Wang

**Affiliations:** 1 State Key Laboratory for Biology of Plant Diseases and Insect Pest, Institute of Plant Protection, Chinese Academy of Agricultural Sciences, Beijing 100000, China; raufabatool@yahoo.com (R.B.); klhe@ippcaas.cn (K.H.); zhangtiantao@ippcaas.cn (T.Z.); sxbai@ippcaas.cn (S.B.); 2 Zhengzhou Fruit Research Institute, Chinese Academy of Agricultural Sciences, Henan Joint International Laboratory of South Asian Fruit and Cucurbit, Zhengzhou 450000, China; umermjawad@yahoo.com; 3 Insect Ecology, Institute of Plant Protection, Jilin Academy of Agricultural Sciences, Changchun 130000, China; wang_yangzhou@163.com; 4 School of Life Sciences, Jilin Agriculture University, Changchun 130000, China; baizhi0607@163.com; 5 School of Agriculture and Biology, Shanghai Jiao Tong University, Shanghai 200000, China

**Keywords:** *Ostrinia furnacalis*, *Beauveria bassiana*, *Trichoderma asperellum*, entomopathogenic fungi, combine application, transcriptomic response

## Abstract

*Ostrinia furnacalis*, is the major pest of maize causing significant yield losses. So far, many approaches have been used to increase the virulence of entomopathogenic fungal isolates. The current study is an attempt to estimate synergistic effect of *Beauveria bassiana* and *Trichoderma asperellum* in order to explore larval immune response through RNA sequencing and differentially expression analysis. In vivo synergism was examined in seven proportions (*B. bassiana: T. asperellum* = 1:1, 1:2, 1:3, 1:4, 4:1, 3:1, 2:1) and in the in vitro case, two inoculation methods were applied: seed coating and soil drenching. Results revealed significant decrease in plant damage and high larval mortality in fungal treatments. Fungal isolates mediated the plant defense by increasing proline, superoxide dismutase (SOD), peroxidase (POD), polyphenol oxidase (PPO) and protease activities. Seed coating method was proved to be the most effective in case of maize endophytic colonization. In total, 59 immune-related differentially expressed genes DEGs were identified including, *cytochrome P450, heat shock protein, ABC transporter, cadherin, peptidoglycan recognition protein (PGRP), cuticlular protein*, etc. Further, transcriptomic response was confirmed by qRT-PCR. Our results concluded that, coculture of *B. bassiana* and *T. asperellum* has the synergistic potential to suppress the immune response of *O. furnacalis* and can be used as sustainable approach to induce plant resistance through activation of defense-related enzymes.

## 1. Introduction

Maize (*Zea mays* L.) is cultivated worldwide and commonly known as queen of cereals for its highest production among all cereal crops. Every year, 960 million tons of maize is being produced throughout the world. However, like other crops, cultivation of maize is also not free from constraints including attack of insects, which can be fatal and can reduce production and grain quality [1]. *Ostrinia furnacalis* (Guenée) (Lepidoptera: Crambidae), usually known as the Asian corn borer (ACB), extends from East and Southeast Asia to the Western Pacific islands [2] and is the most important insect pest of maize in China. It tunnels into the stalks and ears of maize plants resulting yield losses of 10–20% or above. In severe cases no harvest situations may also occur [3].

Chemical pesticides are considered to be the only tool to control insects attacks in case of emergency, however, their indiscriminate use has led to some serious problems, such as environmental pollution, insect resistance and toxicity to nontargeted organisms including natural enemies and humans [4]. Thus, in this scenario biological integrated pest management is rising as an environmental friendly substitute to chemical insecticides [5]. A novel strategy for plant protection is the induction of integral defense mechanisms by prior treatment with biological inducers [6]. Many fungal entomopathogens including *Beauveria bassiana, Lecanicillium lecanii, Metarhizium anisopliae, Isaria fumosorosea* are widely used for plant protection against wide range of insect pests [7,8].

Recent research interests in advancement of microbial fertilizer has been shifting towards the use of cocultures or microbial consortium of different entomopathogenic strains to increase their stability and efficiency in plant growth and biocontrol activities in different environmental conditions [9]. Entomopathogenic fungal hyphae penetrates through cuticle, grows inside the body and kills the target insects. They are considered to be less vulnerable to insect resistance development and have no toxicity against humans and animals [8,10]. Evidence has suggested that, in addition to causing mortality in insects, fungal entomopathogens also improve plants nutrient uptake, stimulate hormone production and increase tolerance to abiotic and biotic stresses thereby, boosting plant growth [11].

*B. bassiana* and *Trichoderma asperellum* are being widely used in different crops as an entomopathogenic integrated pest management and proved to be plant growth enhancers as reported in previous studies [12,13]. Isolation of these fungi from plant tissues, soil and infected insect bodies [14,15] is quite easy. Though *B. bassiana* is more famous for its high microbial control potential, recent research has revealed that it may possess an endophytic lifestyle. Endophytic *B. bassiana* can protect plants against several pests [16,17]. However, very few studies have been performed to understand the effects of colonization by *B. bassiana* on growth of plants and their responses under abiotic stresses [18]. *Trichoderma*, which is a genus of plant-root- and soil- associated fungi among those important biological control agents, represents more than 60% of all known listed species used in infectious plant disease control and which also play important role in growth [13,19].

Plant–insect–microbe interactions and changes induced by microbes not only affect plant defense, growth, and yield but also the fitness of insect herbivores via wide range of direct and indirect mechanisms [20]. Microbes that are associated with insects both nutritional and defensive endosymbionts also including entomopathogens can alter the ability of their insect hosts to exploit host plants. In recent years, more evidences have been recorded that fungal entomopathogens, considered as insect pathogens, can also play potential roles as endophytes, plant disease antagonists and plant growth promoters [21]. Continuous discoveries on the diversity and role of plants and insect-associated microbial communities are stimulating the development of new pest control strategies based on microbe-mediated enhanced plant defenses [22]. It has been reported that entomopathogenic microbes including fungi can stimulate the activity of defensive enzyme (superoxide dismutase (SOD, EC 1.15.1.1), peroxidase (POD, EC 1.11.1.7) polyphenol oxidase (PPO, EC 1.10.3.1) etc.), in plants under pest attack [5].

Insects’ surrounding environments are full of harmful microorganisms and frequent infections are common in such natural environments. So, insects have evolved various defense mechanisms to combat such infectious pathogens. Insect lack adaptive immunity and solely rely on innate immunity for host defense, which is comprised of humoral response including, melanization and synthesis of antimicrobial peptides (AMPs) [23] and cellular response including phagocytosis, encapsulation, or nodulation of pathogens [24]. AMPs are directly cytotoxic to a wide range of microorganism and are synthesized through the signaling pathways “Toll and Imd” [25]. When pathogenic microbial infection occur, insect immediately respond by activating its cellular and humoral responses, through several steps and clear the infection [26]. Pattern recognition receptors (PRRs) recognize the invading pathogen [27] and initiate the activation of signaling pathways through serine proteases [28]. Lastly, the effectors are prompted to combat the pathogens in specific tissues. To counter the insect defense system, pathogenic fungi have also developed their mechanisms. The insect pathogens release a set of enzymes to breach the cuticle [29] and suppress the host immune system, by releasing secondary metabolites during colonization [30].

In the present study, consortium of *B. bassiana* with *T. asperellum* was used for the first time to estimate the virulence of *B. bassiana* OFDH1-5 and *T. asperellum* GDFS1009, in order to determine their synergistic roles against *O. furnacalis*. An RNA-sequencing approach was used to explore and compare immune responses in the mid gut tissues of fungal treated and nontreated *O. furnacalis*. We specially emphasized on the response of immunity related differentially expressed genes. We further validated the expression of differentially expressed genes by quantitative real time qRT-PCR analysis. Current results gave an overview of Asian corn borer midgut in response to fungal strains and useful information for studying the molecular basis of host–entomopathogenic-fungus interaction. Moreover, we observed significant differences in the activities of plants’ defense-related enzymes in response to different treatments.

## 2. Results

### 2.1. In Vitro Pathogenicity Bioassay

**Figure 1:**
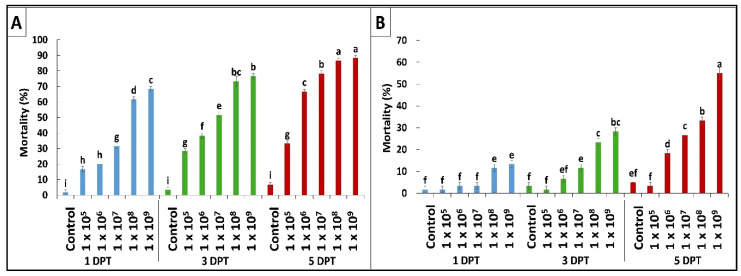
Mortality (%) of *O. furnacalis* larvae fed on *B. bassiana* OFDH1-5 (**A**) and *T. asperellum* GDFS1009 (**B**) incorporated diet at the first, third, and fifth day post treatment (DPT). Means followed by different lowercase letters above each bar indicates significant differences among the treatments (*p* < 0.05).

**Figure 2:**
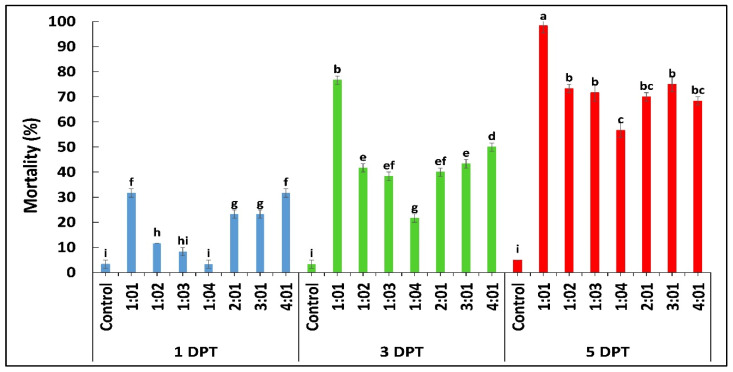
Mortality (%) of *O. furnacalis* larvae fed on diet incorporated with binary combination of *B. bassiana* OFDH1-5 + *T. asperellum* GDFS1009 in different proportions recorded at 1, 3, and 5 days post treatment (DPT). Means followed by different lowercase letters above each bar indicates significant differences among the treatments (*p* < 0.05).

According to the results obtained by diet bioassay, *B. bassiana* OFDH1-5 exhibited significant mortality at all concentrations however, maximum mortality of 68%, 76% and 88% and minimum mortality of 16.7%, 28% and 33% was recorded at 1 × 10^5^ concentration on first, third and fifth day post treatment (Figure 1A). Whereas, mortality rate of *T. asperellum* GDFS1009 was considerably lower as compared to *B. bassiana* OFDH1-5 at all concentrations (Figure 1B). Maximum mortality of only 55% was recorded at the highest concentration (1 × 10^9^) of *T. asperellum* GDFS1009, but when applied as binary combination with *B. bassiana* OFDH1-5 at LC_50_ concentrations (Table 1) in different proportions, a significant increase in mortality was recorded. Maximum mortality (98.3%) was observed when applied in a proportion of 1:1 (*B. bassiana* OFDH1-5: *T. asperellum* GDFS1009) at 5 Days post treatment (DPT). Mortality was relatively low in the case of the 1:4 proportion as compared to others. As the amount of *B. bassiana* OFDH1-5 decrease in combined solution, the mortality rate also falls and vice versa (Figure 2).

### 2.2. Scanning Electron Microscopy

**Figure 3:**
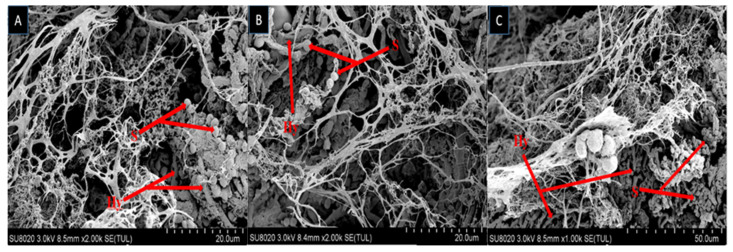
Scanning electron micrograph of infected larva reveals spores and hyphae structures in body cavity; *B. bassiana OFDH1-5* (**A**); *T. asperellum GDFS1009* (**B**) and *B. bassiana OFDH1-5 + T. asperellum* GDFS1009 (**C**). Arrow indicates spores (S) and hyphae (Hy).

**Figure 4:**
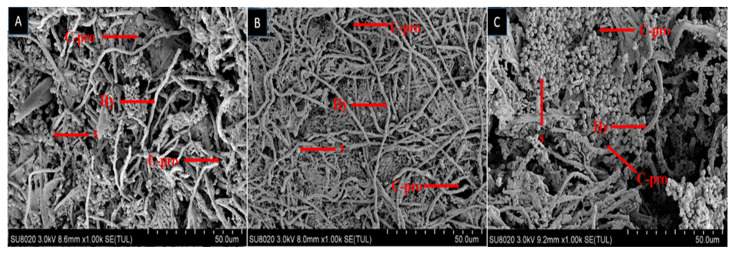
Scanning electron micrograph sowing cuticle proliferation (C-pro) due to hyphal penetration in infected larval body: *B. bassiana* OFDH1-5 (**A**); *T. asperellum* GDFS1009 (**B**) and *B. bassiana* OFDH1-5 + *T. asperellum* GDFS1009 (**C**). Arrow indicates cuticle proliferation (C-pro), spores (S) and hyphae (Hy).

**Figure 5:**
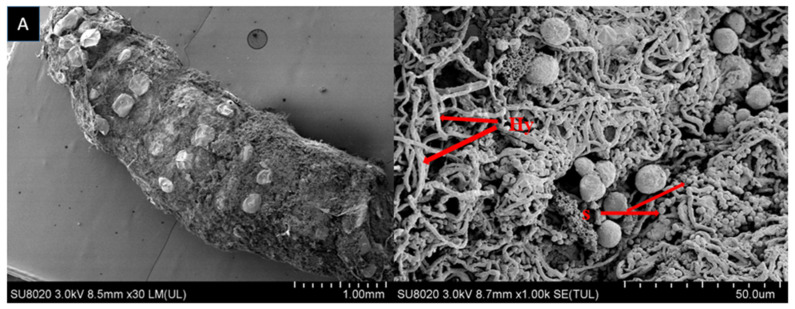

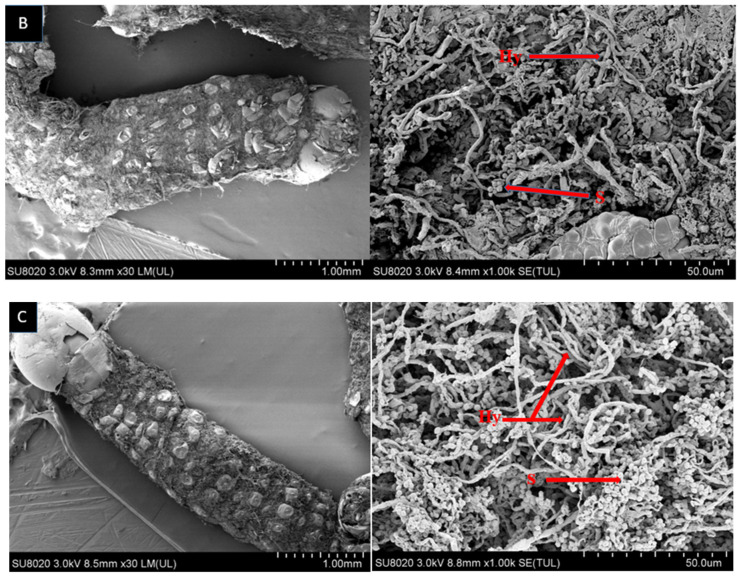
Scanning electron micrograph of infected larval body fully covered with myceli and a closer view showing dense structure of fungal spores and hyphae growing on the body surface: *B. bassiana OFDH1-5* (**A**); *T. asperellum GDFS1009* (**B**) and *B. bassiana OFDH1-5 + T. asperellum GDFS1009* (**C**). Arrow indicates spores (S) and hyphae (Hy).

The growth of fungal spores on dead larvae treated in bioassay were observed by scanning electron microscope (SEM). Mycelium started to grow after larvae was fed on fungal conidia incorporated diet and after 7–10 days the larval body was fully covered with fungal mycelium and spore production started. Scanning electron microscopy of larvae fed on diet incorporated with *B. bassiana* OFDH1-5, *T. asperellum* GDSF1009 and binary combination of both strains clearly revealed the signs of fungal growth on larval body. The spores filled the body cavity of larvae (Figure 3). Proliferation (Figure 4), adhesion, hyphae penetration structures on insect cuticle and fully covered larval body with spores can easily be seen (Figure 5).

### 2.3. Defense Response of Maize Plant

#### 2.3.1. Confirmation of Endophytic Colonization of Plants

By a re-isolation method, white mycelia growth of *B. bassiana* OFDH1-5, green mycelia growth of *T. asperellum* GDSF1009 and mixed greenish white growth from the plants inoculated with binary combination of both strains were observed growing from leaf segments placed in PDA plates after three days whereas, in control treatment, no fungal growth was observed.

#### 2.3.2. Larval Mortality and Maize Plant Damage Rating

Mortality of larvae of *O. furnacalis* was increased significantly due to fungal inoculants whereas, in T7 (plants infested with *O. furnacalis*), the mortality was only 8.7%. Seed coating treatment (T1, T3 and T5) showed maximum mortality (as compared to soil drenching treatment method (T2, T4 and T6). Seed coating with *B. bassiana* OFDH1-5 (T1) and *T. asperellum* GDSF1009 (T3) increased the mortality up to 85% and 53% respectively, whereas, seed coating with binary combination of both strains (T5) increased mortality up to 90%.

The number of tunnels and length of tunnels were also reduced due to fungal inoculation and the maximum tunneling was observed in T7 (insect infestation treatment). Seed coating with binary combination of *B. bassiana* OFDH1-5 and *T. asperellum* GDSF1009 reduced more than 80% of tunnel number and length as compared to insect infestation treatment (T7) (Table 2).

#### 2.3.3. Biochemical Analysis of Maize Plants

##### Antioxidant Enzyme Assay

**Figure 6:**
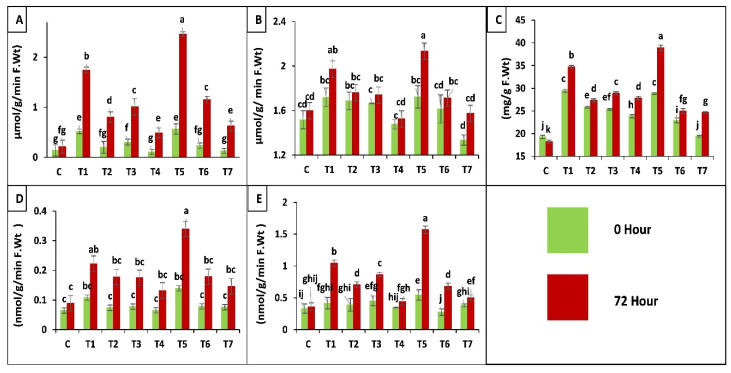
Effect of entomopathogenic fungi on POD (**A**); SOD (**B**); proline (**C**); protease (**D**) and PPO (**E**) activity of plants at 0 h and 72 h of insect attack. Treatment detail: uninoculated and uninfested control (**C**); seed coating with *B. bassiana* OFDH1-5 (T1); soil drenching with *B. bassiana* OFDH1-5 (T2); seed coating with *T. asperellum* GDFS1009 (T3); soil drenching with *T. asperellum* GDFS1009 (T4); seed treatment with binary combination of *B. bassiana* OFDH1-5 + *T. asperellum* GDFS1009 (T5); soil drenching with binary combination of *B. bassiana* OFDH1-5 + *T. asperellum* GDFS1009 (T6); infested control (insect control) (T7). Means followed by different lowercase letters above each bar indicates significant differences among the treatments (*p* < 0.05).

According to results application of both fungal strains separately and in combination increased peroxidase (POD, EC 1.11.1.7) activity before (0 h) and after (72 h) insect attack, when applied as seed treatment. Whereas, a slight increase was observed in case of soil drenching application of fungal suspension as compared to control (C), insect control (T7). A maximum increase of 293-fold was observed in plants treated as seed coating with binary combination of both strains (T5). An increase of 179-fold was observed in plants inoculated by seed treatment of *B. bassiana* OFDH1-5 (T1), whereas, only 62-fold increase was observed in *T. asperellum* GDSF1009 treated as seed coating, plants (T3). Minimum increase was seen in plants of insect control treatment (T7) and plants treated with soil drenching of *T. asperellum* GDSF1009 as compared to plants without insect attack (0 h) (Figure 6A).

#### 2.3.4. Chlorophyll a, b and Carotenoids Contents in Maize Plants

In the case of super oxide dismutase (SOD, EC 1.15.1.1) activity of plants inoculated with binary combination as seed treatment showed maximum increase of 35-fold after insect attack. *B. bassiana* OFDH1-5 inoculation as seed treatment showed 25-fold increase. Whereas, minimal increase of 18-fold was observed in insect control (T7) at 72 h after insect infestation in comparison with T7 at 0 h or control treatment and soil drenching of *T. asperellum* GDSF1009 treatment (T4) as compared to all other treatments (Figure 6B).

##### Proline Content of Leaves

Plants inoculated with binary combination of both strains applied by seed treatment (T5) showed the maximum of 49- and 58-fold increase in proline content, before (0 h) and after (72 h) insect attack respectively, when compared with insect control (T7). Minimal increase was observed by insect control (T7) and soil drenching of binary combination treatment (T6) respectively (Figure 6C).

##### Protease (EC 3.4.21.112) Activity

Protease enzyme production in all treatments with seed coating application method showed maximum increase. Whereas, treatments with soil drenching application method showed a little increase. Maximum increase of 133-fold was observed in T5 (seed coating with binary combination of both strains) after insect attack and seed coating with *B. bassiana* OFDH1-5 increased proline activity in plants by 53-fold when compared with T7 (insect control) (Figure 6D).

##### Polyphenol Oxidase (PPO, EC 1.10.3.1) Activity

A significant increase of 212-fold was observed in plants treated with binary combination of both strains by seed coating (T5) and minimum increase of 35-fold was observed in plants treated with *T. asperellum* GDSF1009 by soil drenching method (T4) as compared to inset control (T7). Seed coating with *B. bassiana* OFDH1-5 (T1) increased PPO activity up to 108-fold. (Figure 6E).

**Figure 7:**
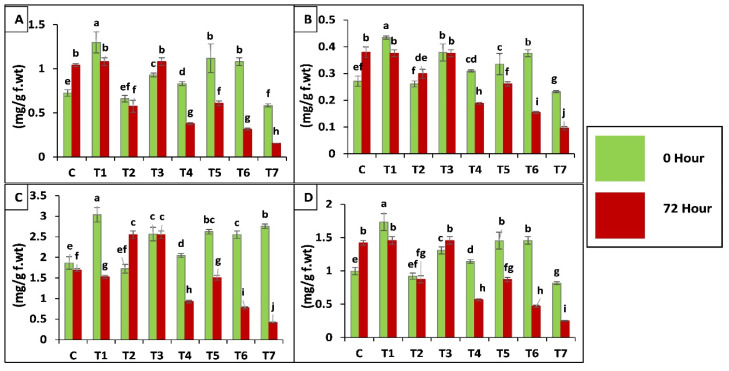
Effect of entomopathogenic fungi on chlorophyll a (**A**); chlorophyll b (**B**); carotenoids (**C**) and total chlorophyll content (**D**) of plants, at 0 h and 72 h of insect attack. Treatment detail: uninoculated and uninfested control (**C**); seed coating with *B. bassiana* OFDH1-5 (T1); soil drenching with *B. bassiana* OFDH1-5 (T2); seed coating with *T. asperellum* GDFS1009 (T3); soil drenching with *T. asperellum* GDFS1009 (T4); seed treatment with binary combination of *B. bassiana* OFDH1-5 + *T. asperellum* GDFS1009 (T5); soil drenching with binary combination of *B. bassiana* OFDH1-5 + *T. asperellum* GDFS1009 (T6); infested control (insect control) (T7).2.4. Transcriptome analysis and identification of differentially expressed genes (DEGs) in ACB. Means followed by different lowercase letters above each bar indicates significant differences among the treatments (*p* < 0.05).

Chlorophyll a, b and carotenoids contents were reduced by 73-, 59- and 84-fold, respectively, in T7 after insect attack as compared to C (control) and inoculated treatments but inoculation with *T. asperellum* GDSF1009 through seed coating significantly increased the chlorophyll content of plants by 79-fold under insect attack as compared to control. Plants inoculated with *B. bassiana* OFDH1-5 (T1) and binary combination (T5, T6) increased the chlorophyll content by 82- and 47-fold respectively before insect attack (0 h) but after insect attack chlorophyll content was found to decrease by 16- and 37-fold respectively in these treatments. Chlorophyll contents of plants inoculated with fungi was observed higher under insect attack as compared to insect control (T7) treatment (Figure 7).

### 2.4. Transcriptome Analysis and Identification of Deferentially Expressed Genes in ACB

**Figure 8:**
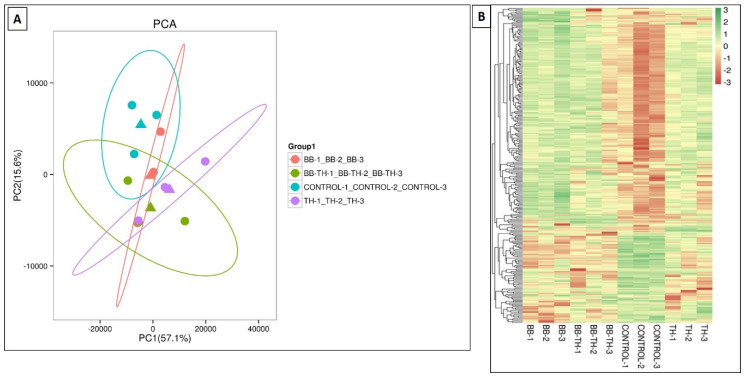
PCA analysis showing variability of data (**A**) and hierarchical cluster analysis showing expression level of DEGs in all treatments (**B**). Green color indicates genes with a higher expression and red color indicates lower expression.

The RNA sequencing data of four treatments *B. bassiana* (BB), *T. asperellum* (TH), *B. bassiana* + *T. asperellum* (BT), and Control after fungal infection revealed total cleaned reads ranging from 42,901,514 to 63,186,052 and in each sample, more than 95.09% of base score Q30 and above. The clean data were mapped to the reference genome, with the mapping ratios varying from 82.58% to 87.07% with GC contents ranging from 48% to 49.46% (Table S1). Our results showed that there were 218 (168—upregulated and 50 downregulated), 38 (13—upregulated and 25 downregulated) and 45 (17—upregulated and 28 downregulated) DEG’s that were identified in Control vs. BB, Control vs. BB+TH and Control vs. TH respectively (Table 3). Variability of data was checked through PCA analysis (Figure 8A). All the DEG’s have been represented in a heat map showing their expressions in response to all the treatments i.e., Control, BB, BB+TH, TH (Figure 8B). Downregulated genes were higher in response to combined treatment (BB+TH) and *T. asperellum* (TH). Venn diagram represents that only eight DEGs were common in all treatments whereas, 188, 18 and 21 DEGs were specifically expressed in Control vs. BB, Control vs. BB+TH and control vs. TH respectively (Figure S1).

#### 2.4.1. Gene Ontology (GO) and Kyoto Encyclopedia of Genes and Genomes (KEGG) Classification and Enrichment Analysis

**Figure 9:**
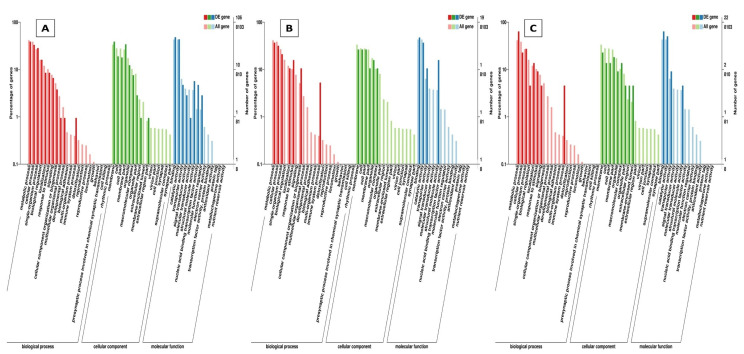
Gene Ontology (GO) classifications of differentially expressed genes. The differentially expressed genes were grouped into three hierarchically stretched GO terms, biological process, cellular component and molecular functions. Control vs *B. bassiana* (Control vs. BB) (**A**); Control vs *B. bassiana + T. asperellum* (Control vs. BB+TH) (**B**) and Control vs *T. asperellum* (control vs. TH) (**C**).

**Figure 10:**
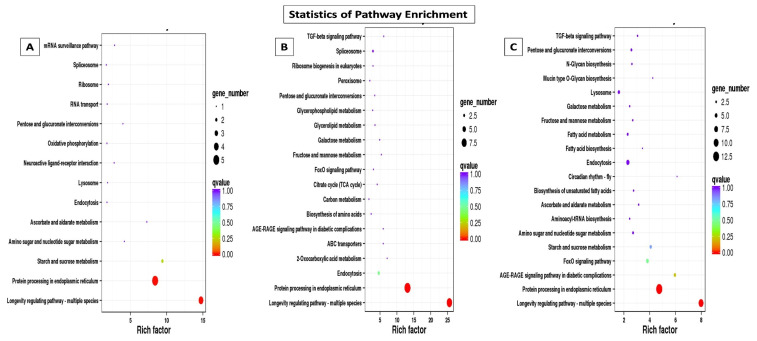
Kyoto Encyclopedia of Genes and Genomes (KEGG) pathway enrichment scatter plot. The vertical axis represents the path name, and the horizontal axis represents the path factor corresponding to the Rich factor. The size of the q-value is represented by the color of the point. The smaller the q-value, the closer the color is to the red color. The number of differential genes included in each pathway are expressed by the size of the point. Control vs *B. bassiana* (Control vs. BB) (**A**); Control vs *B. bassiana + T. asperellum* (Control vs. BB+TH) (**B**) and Control vs *T. asperellum* (Control vs. TH) (**C**).

Through GO annotation, differentially expressed genes were classified in 28 different groups, out of which, 11 belong to biological processes, nine belong to cellular components and eight belong to molecular functions, control vs. *B. bassiana* treatment. In Control vs. BB+TH, DEGs were classified into 17 groups, from which seven belong to biological processes, six belong to cellular components and four belongs to molecular functions. In control vs. *T. asperellum*, DEGs were classified into 21 groups from which eight belong to biological processes, nine belong to cellular components and four belong to molecular functions. In all three treatments the most enriched groups of biological processes were metabolic processes, cellular processes, response to stimulus and immune system processes, in cellular components most dominant groups were cell membrane, cell parts and organelle, and in molecular function binding, signal transducer activity, catalytic activity were the most enriched groups (Figure 9). The KEGG classification analysis also categories DEGs in to 20, 19 and 14 groups in treatments BB, BB+TH and TH respectively, and the topmost enriched groups in all treatments were protein processing in endoplasmic reticulum and longevity-regulating pathways (Figure 10).

#### 2.4.2. Expression Pattern of Immune Related Genes and qRT-PCR Validation

By screening differentially expressed genes data we identified a total of 59 immunity-related genes, 39 in Control vs. BB, 13 in Control vs. BB+TH and six in Control vs. TH, which include *peptidoglycan recognition, cytochrome P450, heat shock proteins, signal transducers, longevity regulators, ACB transporters, cuticular protein, chitinase, UDP-glucuronosyltransferase* and *cadherin* (Table 4). We identified two cuticle protein genes which were downregulated in Control vs. BB and Control vs. BB+TH whereas up regulated in Control vs. TH. Two PGRP’s were identified, PGRP-s gene-LOC114352122 was upregulated in all treatments and PGRP-B gene-LOC114352113 was upregulated only in Control vs. BB but downregulated in Control vs. TH and Control vs. BB+TH. Two ABC transporter genes which are associated with drug resistance, were identified and both were upregulated in all treatments as compared to control. Cytochrome P450 gene was downregulated in all treatments as compared to control and two heat shock proteins, HSP70 and HSP 68, were downregulated.

**Figure 11:**
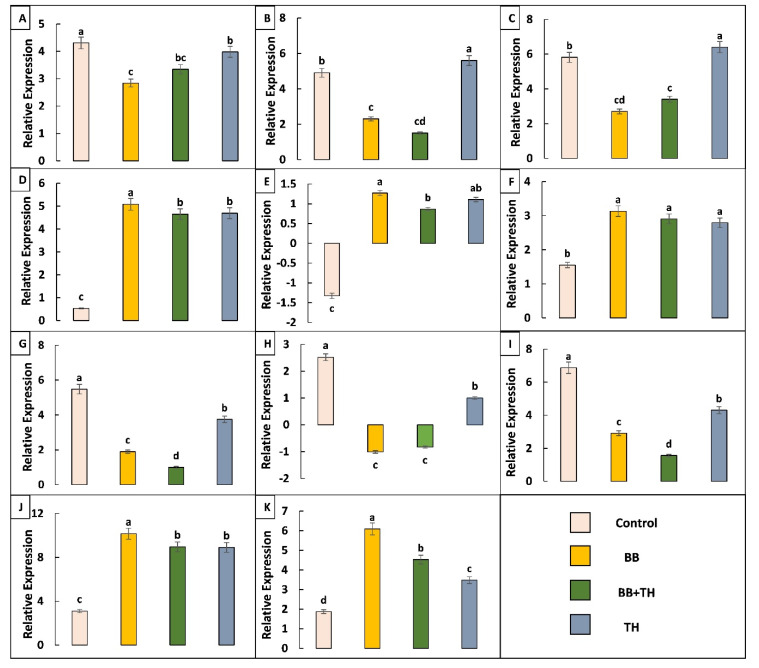
The qRT-PCR analysis of selected differentially expressed genes to confirm expression patterns indicated by RNA-sequencing. UDP-glucuronosyltransferase gene-LOC114350074 (**A**); larval/pupal rigid cuticle protein gene-LOC114366353 (**B**); larval/pupal rigid cuticle protein 2 gene-LOC114366367 (**C**); ABC transporter 1 gene-LOC114354308 (**D**); ABC transporter 2 gene-LOC114353130 (**E**); cadherin gene-LOC114362562 (**F**); HSP70 gene-LOC114356221 (**G**); HSP68 gene-LOC114363773 (**H**); cytochrome P450 gene-LOC114353087 (**I**); PRPB gene-LOC114352113 (**J**) and PRPS gene-LOC114352122 (**K**). Means followed by different lowercase letters above each bar indicates significant differences among the treatments (*p* < 0.05).

From differentially expressed gene data, 11 genes related to larval resistance and immunity were selected and analyzed through qRT-PCR. From qRT-PCR analysis, all the tested genes showed same trend of expression as in transcriptome results, some of the selected genes showed different trend in different treatments (Figure 11).

## 3. Discussion

Nowadays, biological pest control approaches are of great interest thus, by using entomopathogenic fungal strains with enhanced efficiency serves as an effective and safe alternative for chemical insecticides [4]. The biocontrol agents’ modes of action include inhibition or parasitism of pathogens, by using antibiotics often in combination with extracellular cell wall degrading enzymes; competition for nutrients (i.e., iron, nitrogen or carbon) in colonization sites; stimulation of plant resistance mechanisms [31,32]. In recent years, the research interest is shifting towards the use of coculture techniques by mixing two or more biocontrol agents to increase their efficiency, so, the current study comprehended the determination of virulence ability of *B. bassiana* OFDH1-5, *T. asperellum* GDFS1009 and synergistic effects of both strains in different proportions against *O. furnacalis*. Defense response of plants induced by fungal inoculants was also studied. According to the results of in vitro virulence bioassay, both strains were found to be an effective controlling agent against *O. furnacalis* but the effectiveness of *B. bassiana* OFDH1-5 was higher than *T. asperellum* GDFS1009 at all concentrations used during this research. Binary combination (1:1) of both fungi showed highest mortality of 98.3%. Mortality was seen to be increased with the increase of conidia and time. Highest mortality was recorded at fifth day post treatment. The results are in accordance with the previous findings of [33], who reported the efficiency of entomopathogenic strains and binary combination techniques for insect control. Ref. [34] also reported that coculture of *T. asperellum* GDFS1009 with *Bacillus amyloliquefaciens* enhanced the biocontrol and plant growth activity. According to previous studies, great success has been achieved in controlling *O. furnacalis* and pine caterpillars by application of fungal bio-control agents in China, for example *B. bassiana* [35]. Usually the virulence of entomopathogenic fungi and mortality of targeted insect depends upon the concentration, exposure time and temperature [36]. Unlike bacteria and viruses, fungi can also kill sucking insects including mosquitos and aphids along with chewing insects because they can directly penetrate the host cuticle and infect the insect body [37].

Fungal infection begins when the conidia attach to the insect body, starts to germinate and produce hyphae then they release toxins to destroy the immune system of insect. Afterwards, the hyphae penetrate through cuticle towards outside to cover the whole body from outside and cover the body with white or green spores [38]. Scanning electron microscopy revealed the infected larval body fully covered with fungal growth. Adhesion, penetration and proliferation structures of *B. bassiana* OFDH1-5 and *T. asperellum* GDFS1009 were observed in the current research. The dense network of spores and hyphae was also visible in the body cavity of larvae. These observations match with the findings of Gabarty, Salem, Fouda, Abas and Ibrahim [38] and Asensio, et al. [39] who observed that *Lecanicillium dimorphum* and *Lecanicillium* cf. psalliotae made infection structures on scale insects. Enzymes production by biocontrol agents play an important role in killing pathogens. Infection by *B. bassiana* has been shown to require direct penetration of the insect host integument by growing hyphae, apparently facilitated by both mechanical and enzymatic activity [40,41]. The biocontrol mechanisms of *Trichoderma* include antibiotics, competition and mycoparasitism [42]. Mycoparasitism of *Trichoderma* is a complex process including the secretion of cell-wall-degrading enzymes (CWDEs) [43]. The growth of fungal hyphae is facilitated by many enzymes associated with conidia and their primary function is to hydrolyze the epicuticular wax layer of insect body and provide nutrients which are required for the formation of germ tube. Wagner and Lewis [44], reported that, vanishing of wax layer beneath the appressoria of *Metarhizium anisopliae* on the cuticle of wireworm indicates enzymatic activity.

Plants provides a suitable platform to microorganisms so that they can play their roles in plant growth promotion and disease suppression [45]. After in vitro prescreening, a pot experiment was performed to estimate the ability of *B. bassiana* OFDH1-5 and *T. asperellum* GDFS1009 to induce defense and to observe biochemical and physiological response of maize plants against the Asian corn borer attack. For this, two types of treatment methods were applied (seed coating and soil drenching). Endophytic colonization of fungal isolates in plants were detected by a reisolation technique and all were successfully isolated from seed inoculated treatments whereas, the re-isolation percentage from soil drenching application treatments was relatively low. Plant colonization largely depends upon type of inoculation method and seed inoculation or seed coating method resulted in superior colonization as compared to other methods. Moreover, endophyte inoculation at seed stage could have the advantage of colonizing both seed radical and the plumule, which are close to one another in the seed Muvea, et al. [46]. Studies have shown that *B. bassiana* when applied to the maize plants, it colonizes and moves within the plant [47]. After *B. bassiana* penetrates maize, the primary hyphae develop rapidly into a branched, multicellular mycelial network. Hyphae may grow directly into neighboring epidermal cells and subtending palisade parenchyma and grow into intercellular spaces [48]. Previous study by Umadevi, et al. [49] demonstrated the endophytic colonization of black pepper by *Trichoderma* species. Muvea, Meyhöfer, Subramanian, Poehling, Ekesi and Maniania [46], reported that different *Trichoderma* isolates including *T. harzianum* and *T. asperellum* can effectively colonize various parts of onion plant and have antagonistic effect against onion thrips.

In the current study, the mortality of *O. furnacalis* larvae was recorded to be increased significantly in *B. bassiana* OFDH1-5 inoculated treatments as compared to insect-infested treatment (T7). Similarly, fungal inoculants were found to be pathogenic against *O. furnacalis* and decreased the number and length of tunnels as compared to infested treatment only (T7). Hardy, et al. [50] stated that endophytic fungal growth could protect several plants from herbivore attack. The presence of *B. bassiana* in plant tissue may affect insects that ingest the fungus [51]. *B. bassiana* has been used as a biological insecticide to control a wide variety of pests including aphids [51] and corn borers [44]. *T. asperellum* GDFS1009 was not found to be a very strong agent to kill *O. furnacalis* larvae and caused the mortality of only 53.3% whereas, when applied as coculture with *B. bassiana*, the mortality was increased. These findings were supported by Shakeri and Foster [52] who stated that *Trichoderma* strains did not effectively control borer larvae, but their activity can be increased by transforming its original chitinase gene and it was stated by [53] that cocultivation of two biocontrol agents can act as inducer in activating silent gene through competition and intercommunication.

Herbivorous pest attack induces an osmotic imbalance and oxidative stress in plants which was adopted by plants through increasing antioxidant enzyme production and proline content [5]. In the scavenging process of reactive oxygen species (ROS), superoxide dismutase is the first enzyme, and in this study, it was significantly stimulated in plants infested by *O. furnacalis*. A similar increase was also observed in plants inoculated with fungal strain under unstressed conditions whereas, plant seed inoculated with *B. bassiana* show more than 100-fold increase. The highest increase of more than 200-fold was observed for SOD (EC 1.15.1.1) in plants inoculated by seed coating with binary combination of *B. bassiana* and *T. asperellum*. A similar trend of increase was observed in peroxidase (POD, EC 1.11.1.7) enzyme production among all treatments. The enhanced scavenging action of SOD results in the production of H_2_O_2_, which was detoxified by POD and it can also act as a signaling agent to induce defense genes as reported by Bano and Muqarab [5].

Proline serves as an energy source in plants and is also known as hydroxyl radical scavenger [54]. Maximum increase in proline content under stressed and unstressed conditions was observed in seeds coated with binary combination of both strains. Mansour [55] reported that biotic and abiotic stresses can cause the accumulation of proline in plants.

Polyphenol oxidase (PPO, EC 1.10.3.1), which helps in oxidation of polyphenols into quinones, is involved in lignification of plant cells during insect attack. It also plays an important role in activating defense reactions and inducing resistance in plants against insect attack [56]. An increase in PPO was observed in all plants infested with *O. furnacalis* which was further augmented by fungal inoculants. More than a 100-fold increase in PPO production was observed in plant seeds coated with a binary combination of both fungi as reported by Bano and Muqarab [5] who stated that microbial inoculation augmented the production of PPO in stressed plants as compared to non-stressed plants.

Protease activity (EC, 3.4.21.112) was observed to be increased by insect infestation and is further increased in inoculated plants under stress conditions, whereas maximum increase of protease was also observed in plant seed coated with a mixture of both fungi. Proteases play a defensive role against predators and pathogens [57]. The results were in accordance with Joe and Muthukumaran [58], who reported that higher activities of PPO and protease results in a decreasing feeding activity and causes lower growth rate of *S. Litura* in tomato. Photosynthesis is one of the most important processes in plants. Chlorophyll content of plants was found to be increased significantly in fungal inoculation treatments whereas, insect infestation negatively affected chlorophyll content in all treatments but fungal inoculation improved chlorophyll content as compared to stressed plant as reported by [59,60] Decrease in chlorophyll content is the indication of photo-oxidation and has been previously reported by Rahdari, et al. [61]. When oxidative damage occurs in plant due to stress conditions, several enzymatic (SOD, POD, CAT) and non-enzymatic (ascorbate, carotenoids, phenolic compounds etc.) antioxidants concentrated in chloroplast get activated and carotenoids are one of the non-enzymatic antioxidants present in substantial amounts to protect plants by scavenging reactive oxygen species [62].

Considering the potential results and importance of *B. bassiana* OFDH1-5, *T. asperellum* GDFS1009 and their combined application in control of *O. furnacalis*, a transcriptome-based analysis of immune response of *O. furnacalis* after fungal infection was performed using illumina sequencing. By screening differentially expressed genes data, we identified a total 59 of immunity related genes, 39 in Control vs. BB, 13 in Control vs. BB+TH and seven in Control vs. TH including, *peptidoglycan recognition, cytochrome P450, heat shock proteins, signal transducers, longevity regulators, ACB transporters, cuticular protein, chitinase, UDP-glucuronosyltransferase* and *cadherin*. During insect defense against pathogens, the cuticle is the first barrier which along with protecting also maintains shape and mobility of insect [63,64]. *Chitin* and *cuticle proteins* are the major components of insect cuticle [64]. They contribute in drug resistance, stress resistance, and insect immunity. When insects experience harsh environmental conditions or pathogenic attack, genes encoding *cuticle proteins* induce to stabilize and strengthen cuticular structure, provide resistance and maintain insect survival [65,66]. According to [67], gene encoding *cuticle protein* B*mcb10* is significantly upregulated against bacterial infection. Ref. [68] also stated that *cuticle protein* can perform a wound-healing role in *Anopheles gambiae* adult and larvae. In the current study, we identified two *cuticle protein* genes which were downregulated in Control vs. BB and Control vs. BB+TH whereas they were upregulated in Control vs. TH. The cuticle gene, in response to pathogen attack, can transmit exogenous adverse stimulation and activate the process of melanization. Through our results it is clear that insect immune system become active against *T. asperellum* attack whereas, *B. bassiana* alone and in combination with *T. asperellum* decrease the immune response and reduced ACB survival. *Isaria fumosorosea* release *chitinase, chitosanase, lipase*, to physically penetrate the host and suppress its regulatory system, and a beauvericin compound to paralyze the host [69]. Another group of proteins known as *pattern-recognition proteins* play an important role in recognition of invading microorganisms. They include *peptidoglycan recognition proteins (PGRPs), b-1,3-glucan recognition protein (bGRPs)*/*gram-negative binding proteins (GNBPs), C-type lectins (CTLs), scavenger receptors (SCRs)* [24]. In the present study, we identified two PGRP’s. The *PGRP-s gene-LOC114352122* was upregulated in all treatments and *PGRP-B gene-LOC114352113* was upregulated only in Control vs. BB but downregulated in Control vs. TH and Control vs. BB+TH. Previously, Ref. [70] reported that *PGRPs* were downregulated in *D. melanogaster* when injected with *M. anisopliae* whereas, in contrast, Ref. [24] and [71] stated the upregulation of PGRPs in response to *B. bassiana* and *M. acridium*. The first *PGRP* was isolated as a pattern-recognition receptor to trigger a polyphenoloxidase (PPO) activating cascade from silkworm hemolymphs [72], and [24] concluded from this study that *O. furnacalis PGRP* can act as a peptidoglycan receptor in activating PPO cascade under *B. bassiana* attack.

Two *ABC transporter* genes which are associated with drug resistance, were identified in our study and both were upregulated in all treatments as compared to control. These membrane bound transporters are linked with solutes movements through the lipid membranes. In previous studies, *ABC transporters* were associated with Bt resistance in the midgut of Cry1Ab- and Cry1Ac-resistant larvae [73,74]. Ref. [75] also reported differentially expressed *ABC transporters*, which play a critical role in resistance.

The mechanism of insecticide detoxification occurs in all insect species, which involves various enzymes encoded by the *cytochrome P450* family [76]. *Cytochrome P450*, being an important class of enzymes, is involved in metabolism of xenobiotics including drugs, plant secondary metabolites and pesticides, and endogenous substances [10]. In the current study, we identified that the *cytochrome P450* gene was downregulated in all treatment compared to control. Ref. [77] in their study stated that the overexpressed P450 gene may improve the detoxification capability of the Asian corn borer against flubendiamide and can be involved in *O. furnacalis* resistance, whereas we observed that *P450* was downregulated indicating the detoxification mechanism of *O. furnacalis* was suppressed due to entomopathogenic fungi.

Genes, including *heat shock proteins*, were downregulated, whereas *cadherin* was upregulated in all treatments. *HSP 70* [78] and *Cadherin* were reported as toxin-binding receptors in many previous studies [75,79]. To counter the insect defense system, pathogenic fungi have also developed their own mechanism, in which they use a set of enzymes to puncture the insect cuticle [29] and suppress the insect immune system by releasing secondary metabolites during colonization [30,80]. When toxins interact with *cadherin*, it initiates proteolytic cleavages that prompt the toxin oligomerization, which binds with secondary receptors. After binding, these oligomers insert in the membrane and form a pore to make the membrane more permeable. Lastly, these pores create osmotic shock leading to the death of cell [75,81]. In insects, environmental stress prompts the expression of different proteins like *heat shock proteins* [10] but, in our study, the transcriptional level two *heat shock proteins*, *HSP70* and *HSP68*, were downregulated. It can be stated that entomopathogenic fungal injection in the larval body suppresses its defense mechanism by suppressing the activity of several immune- or resistance-related genes. Ref. [75] also stated in his study that *heat shock protein* was downregulated in susceptible and upregulated in Bt resistant strain of Asian corn borer.

In the current study, the seed coating or seed inoculation method of inoculation were found to be more effective in colonizing plants, controlling *O. furnacalis*, and enhancing plant defense enzyme activities in maize plants as compared to the soil drenching method of inoculation. Seed inoculation could be advantageous in terms of low inoculum requirement as compared to augmentative sprays [82]. Further, seed treatment could provide opportunities for endophytic fungi colonization at the young seedling stage for early protection and enhanced seedling health. Backman and Sikora [83] outlined that integrated pest management on seeds reduces costs and environmental impact, while allowing the biological agent to build up momentum for biological control. The broad array of endophyte-induced defense mechanisms in plants against insect pests such as production of toxic or distasteful chemicals [84] and pathogenic interaction to insects [85] could decrease insect fitness. Moreover, when two biocontrol agents were used together to obtain joint action against pest attack, one acts as a stress inducer and other acts as control agent, and they work more effectively against herbivory attack, but there are certain biotic and abiotic factors that can affect the combined activity of biocontrol agents like soil type, condition of host plants, temperature, etc. [86]. A study by [87] stated that combined application of biocontrol agents by different mechanisms can effectively control the incidence of *Duponchelia fovealis* in strawberry plants. The advantages of fungal BCAs in economic mass production, easy-to-use formulation, sustainable control efficacy and environmental safety suggest a bright future of mycoinsecticides and mycoacaricides not only in China but also in the world.

## 4. Material and Methods

### 4.1. Source of Insect and Fungal Isolates

*O. furnacalis* larvae used in this study were obtained from Institute of Plant Protection, Chinese Academy of Agricultural Sciences, Beijing, China. The larvae were reared on artificial diet at 27 ± 2 °C, 60–80% relative humidity with a photoperiod of 16:8 h light: dark (L:D). *Beauveria bassiana* OFDH1-5 (preservation number: ACCC32726) was obtained from Jilin Academy of Agricultural Sciences, Gongzhuling, Jilin Province, China. *Trichoderma asperellum* GDFS1009 (Accession number: JQ617308 for *tef1* and JQ617295 for ITS) was provided by School of Agriculture and Biology, Shanghai Jiao Tong University, Shanghai, China.

### 4.2. Conidial Suspension Preparation

Fungal strains were grown in sterile petri dishes containing Potato Dextrose Agar (PDA) medium and incubated for 15 days at 25 ± 2 °C. For preparation of conidial suspension, the spore powder from freshly prepared plates were harvested by scrapping and suspended in sterile distilled water containing 0.1% tween 80 (*v*/*v*). At last, suspension was shaken vigorously in rotatory shaker for 5 min to make a uniform conidial suspension. Conidial concentration was determined with the help of Neubauer haemocytometer [88]. Five different concentrations (1 × 10^5^, 1 × 10^6^, 1 × 10^7^, 1 × 10^8^, and 1 × 10^9^) of conidial suspension were prepared by serial dilution. A germination test was performed prior to bioassay by the method described by Yeo, et al. [89].

### 4.3. In Vitro Pathogenicity Bioassay

The virulence of *B. bassiana* OFDH1-5, *T. asperellum* GDFS1009 and binary combination of their LC_50_ (lethal concentration causing 50% mortality) (Table 1) in seven different proportions (BB-OFDH1-5: TH-GDFS1009 = 1:1, 1:2, 1:3, 1:4, 2:1, 3:1, 4:1) against *O. furnacalis* was estimated by diet incorporated method described by Leckie [51] with slight modifications. LC-50 value of both strains was calculated by Probit analysis using Polo Plus (Version 1.0, LeOra Software, Berkeley, CA, USA) [90]. An artificial diet was mixed with conidial suspension (diet: Spore suspension = 1:1.2 *w*/*v*) to form a testing medium. For control treatment sterilized 0.1% (*v*/*v*) tween 80 was used [91]. An equal amount of diet was added in 31.4 cm^3^ plastic container and one second instar larvae was placed in it. Each treatment had 20 replicates with one insect each. All containers were kept at 25 ± 2 °C and 60–80% relative humidity with a photoperiod of 16:8 h light: dark (L: D). Larval mortality percentage was calculated at 1, 3 and 5 days post treatment (DPT).

### 4.4. Scanning Electron Microscopy (SEM)

Scanning electron microscopy was performed to detect the growth of fungal isolates on larval bodies. For sample preparation, the dead larvae were placed in sterilized petri dish lined with moist filter paper and incubated at 25 ± 2 °C and 70–80% relative humidity for seven days to facilitate the proper growth of fungal spores over larval body. After that, the larvae were fixed in 2.5% glutaraldehyde for 24 h followed by sample drying by a CO_2_ critical point dryer (Tousimis—Autosamdri—825). Then they were coated by gold Sputer coater (HITACHI MC 1000). Finally the samples were examined by scanning electron microscope (HITACHI SU 8020) [38].

### 4.5. Defense Response of Maize Plant

A pot experiment was performed to study the response of defense related enzymes and photosynthetic pigments in *B. bassiana* OFDH1-5 and *T. asperellum* GDFS1009 treated plants before and after infestation with *O. furnacalis*. The experiment was performed in May 2019 at Jilin Academy of Agricultural Sciences, Gongzhuling, Jilin Province, China in a completely randomized design (CRD) with factorial arrangements. Maize seeds of variety ‘Jingke 968′ were surface sterilized by washing with 95% (*v*/*v*) ethanol for 1–2 min followed by dipping in 0.2% (*w*/*v*) HgCl_2_ solution for 3 min and then washed three times with sterilized distilled water [5].

#### 4.5.1. Application of Fungal Suspensions and Sowing

Conidial suspension of *B. bassiana* OFDH1-5 (1 × 10^9^), *T. asperellum* GDFS1009 (1 × 10^9^) and combined suspension of *B. bassiana* OFDH1-5 +*T. asperellum* GDFS1009 (5.9 × 10^5^+ 8.4 × 10^8^) in proportion of 1:1 was prepared as described earlier. Two methods of inoculation were applied: seed coating and soil drenching. For seed coating, surface sterilized seeds were soaked in conidial suspension and placed in a shaker incubator (HZP-250) at 25 ± 2 °C and 200 rpm for 12 h [92]. Seeds were dried under a lamina flow hood [93]. For control and *O. furnacalis* treatment, seeds were soaked in sterilized 0.1% (*v*/*v*) tween 80, and for soil drenching treatments, the seeds were dipped in sterile distilled water. Treated and untreated seeds were sown in plastic pots (30 cm in diameter and 41 cm in length) filled with autoclaved soil and sand (3:1). The soil drenching method of inoculation was applied after one week of germination by pouring 30 mL conidial suspension per plant in the soil around root zone. Treatments applied were C (untreated Control), T1 (seed dressing with *B. bassiana* OFDH1-5 and infested with *O. farnicalis*), T2 (soil drenching with *B. bassiana* OFDH1-5 and infested with *O. farnicalis*), T3 (seed dressing with *T. asperellum* GDFS1009 and infested with *O. farnicalis*), T4 (soil drenching with *T. asperellum* GDFS1009 and infested with *O. farnicalis*), T5 (seed dressing with combined conidial suspension of *B. bassiana* OFDH1-5 and *T. asperellum* GDFS1009 *and* infested with *O. farnicalis*), *T6* (soil drenching with combined conidial suspension of *B. bassiana* OFDH1-5 + *T. asperellum* GDFS1009 and infested with *O. farnicalis*), *T7* (infested with *O. furnacalis* only). Each treatment had 10 replicates with one plant per pot.

#### 4.5.2. Confirmation of Endophytic Colonization of Plants

Endophytic colonization of maize plants by inoculated fungi was confirmed by a reisolation method. Leaves were surface-sterilized in 1% (*v*/*v*) sodium hypochlorite for 3 min, 70% (*v*/*v*) ethanol for few minutes and then washed with sterile distilled water three times and placed on sterilized filter paper. Leaves were cut into small segments by using sterilized scissors and placed on potato dextrose agar plates. After incubation of three to seven days at 25 ± 2 °C, the presence or absence of fungal isolates was recorded [48].

#### 4.5.3. Insect Infestation and Sampling

At V5 stage, 0 h samples (before insect attack) were collected from each treatment and flash frozen in liquid nitrogen till further use. Five second-instar larvae of *O. furnacalis* were placed at the whorl of each plant and allowed to feed freely. After 72 h the leaf samples around the feeding areas were collected and immediately flash frozen in liquid nitrogen for physiological and biochemical analysis [5].

#### 4.5.4. Larval Mortality and Maize Plant Damage Rating

When fifth-instar larvae were observed on plants, they were harvested, stalk was dissected and number of tunnels, tunnel length, dead and surviving larvae were recorded [94].

#### 4.5.5. Physiological and Biochemical Analysis of Plants

##### Antioxidant Enzyme Assay

Peroxidase (POD, EC 1.11.1.7) estimation was done as described earlier by Reddy, et al. [95] with minor modifications. One gram of plant material was homogenized in 10 mL potassium phosphate buffer (PH 7.0) and centrifuged at 10,000 rpm for 10 min at 4 °C. Clear supernatant was collected, and optical density was recorded at 430 nm for 3 min by adding 0.5 mL of 1% (*v*/*v*) H_2_O_2_ in clear supernatant.

Superoxide dismutase (SOD, EC 1.15.1.1) activity was conducted by using an already established method by Beauchamp and Fridovich [96] and absorbance was recorded at 560 nm.

##### Proline Content of Leaves

Proline content of leaves was estimated according to the protocol of Bates, et al. [97]. The absorbance was measured at 520 nm using spectrophotometer and toluene was taken as a blank. The formula used to calculate proline content is:

$$Proline\;\left(\mu g/g\right)=\frac{\left(k\;value\;\times \;dilution\;factor\;\times \;absorbance\right)}{Sample\;weight}$$

Where, k value=17.52 and dilution factor=2

##### Protease (EC 3.4.21.112) Activity

Protease activity was performed by the method of McDonald and Chen [98], in which 100 mg of leaf sample was incubated at 30 °C with 4 mL of 1% (*w*/*v*) casein (in citrate buffer pH 7.0) for one hour. Then 5 mL of trichloroacetic acid was added and the precipitate was allowed to settle down for 30 min. The content was filtered through Whatman No. 40 filter paper. After filtration, 1 mL of aliquot of the filtrate was mixed with 5 mL of alkaline reagent mixture which was prepared by mixing 100 mL of sodium carbonate 2% (*w*/*v*), 1 mL of sodium potassium tartrate 2.7% (*w*/*v*) and 1% (*w*/*v*) copper sulphate, then 2 mL of 1 N sodium hydroxide was added. After 10 min, 0.5 mL of Folin phenol reagent was added and mixed. After 30 min, absorbance of the blue color produced was measured at 660 nm.

##### Polyphenol Oxidase (PPO, EC 1.10.3.1) Activity

The method of Kar and Mishra [99] was followed to measure polyphenol oxidase activity of plants with some modification. The reaction mixture was prepared by adding 25 mM potassium phosphate buffer (pH 6.8), 0.1 mL enzyme extract and 0.1 M pyrogallol A. The absorbance of the reaction mixture formed was recorded at 420 nm.

#### 4.5.6. Chlorophyll Content of Plants

Photosynthetic pigments (chlorophyll *a*, *b* and carotenoid) were determined according to Saeidi and Zabihi-e-Mahmoodabad [100]. A mass of 0.1 g of the fresh leaf was homogenized in 6 mL of 80% (*v*/*v*) acetone, and then centrifuged for 10 min at 6000 rpm. The supernatant was collected to record the absorbance at 645, 663 and 470 nm. Eighty percent (*v*/*v*) acetone was used as a blank.

### 4.6. Transcriptome Analysis

#### 4.6.1. Sample Collection and Preparation

The gut of alive fifth instar larvae was extracted after being fed on a fungal incorporated artificial diet for one week. Five larval guts were collected as one biological replicate for each treatment. Three biological replicates were collected and used for gene expression profile analysis and three biological replicates for qRT-PCR analysis. All samples were stored at −80° till further analysis [75].

#### 4.6.2. Library Preparation and Illumina Sequencing

A total amount of 1 μg RNA per sample was used as input material for the RNA sample preparations. Sequencing libraries were generated using NEBNext UltraTM RNA Library Prep Kit for Illumina (NEB, Ipswich, MA, USA) following manufacturer’s recommendations and index codes were added to attribute sequences to each sample. Briefly, mRNA was purified from total RNA using poly-T oligo-attached magnetic beads. Fragmentation was carried out using divalent cations under elevated temperature in NEBNext First Strand Synthesis Reaction Buffer (5×). First-strand cDNA was synthesized using random hexamer primer and M-MuLV Reverse Transcriptase. Second-strand cDNA synthesis was subsequently performed using DNA polymerase I and RNase H. Remaining overhangs were converted into blunt ends via exonuclease/polymerase activities. After adenylation of 3′ ends of DNA fragments, the NEBNext Adaptor with hairpin loop structure was ligated to prepare for hybridization. In order to select cDNA fragments of preferentially 240 bp in length, the library fragments were purified with the AMPure XP system (Beckman Coulter, Beverly, MA USA). Then 3 μL USER Enzyme (NEB, Ipswich, MA, USA) was used with size-selected, adaptor-ligated cDNA at 37 °C for 15 min followed by 5 min at 95 °C before PCR. Then PCR was performed with Phusion High-Fidelity DNA polymerase, Universal PCR primers and Index (X) Primer. At last, PCR products were purified (AMPure XP system) and library quality was assessed on the Agilent Bioanalyzer 2100 system. The clustering of the index-coded samples was performed on a cBot Cluster Generation System using TruSeq PE Cluster Kit v4-cBot-HS (Illumia, Inc., San Diego, CA, USA) according to the manufacturer’s instructions. After cluster generation, the library preparations were sequenced on an Illumina platform and paired-end reads were generated.

#### 4.6.3. Assembly and Functional Annotation

Raw data (raw reads) of fastq format were firstly processed through in-house perl scripts. In this step, clean data (clean reads) were obtained by removing reads containing adapter, reads containing ploy-N and low-quality reads from raw data. At the same time, Q20, Q30, GC-content and sequence duplication level of the clean data were calculated. All the downstream analyses were based on clean data with high quality. Raw sequences were transformed into clean reads after data processing. These clean reads were then mapped to the reference genome sequence. Only reads with a perfect match or one mismatch were further analyzed and annotated based on the reference genome. Hisat2 tools soft were used to map with reference genome. Gene function was annotated based on the following databases: Nr (NCBI nonredundant protein sequences), Nt (NCBI nonredundant nucleotide sequences), Pfam (Protein family), KOG/COG (clusters of orthologous groups of proteins, Swiss-Prot (a manually annotated and reviewed protein sequence database), KO (KEGG Ortholog database), GO (Gene Ontology) [101].

#### 4.6.4. Differential Expression Analysis

A pairwise comparison between libraries of all treatments with control group was carried out for the identification of DEGs in response to *B. bassiana* and *T. asperellum* infection. Differential expression analysis of two conditions/groups was performed using the DEseq. DEseq provide statistical routines for determining differential expression in digital gene expression data using a model based on the negative binomial distribution. The resulting *p* values were adjusted using the Benjamini and Hochberg’s approach for controlling the false discovery rate. Genes with an adjusted *p*-value < 0.05 found by DEseq were assigned as differentially expressed [102].

#### 4.6.5. Go and KEGG Pathway Enrichment Analysis

Gene Ontology (GO) enrichment analysis of the differentially expressed genes (DEGs) was implemented by the GOseq R packages based Wallenius noncentral hypergeometric distribution [103], which can adjust for gene length bias in DEGs. KEGG [104] is a database resource for understanding high-level functions and utilities of the biological system, such as the cell, the organism and the ecosystem, from molecular-level information, especially large-scale molecular datasets generated by genome sequencing and other high-throughput experimental technologies (http://www.genome.jp/kegg/). We used KOBAS [105] software to test the statistical enrichment of differential expression genes in KEGG pathways.

#### 4.6.6. Validation of Defense Related DEG’s by RT-qPCR

To validate the expression level exhibited by transcriptome data of randomly selected defense related genes, real time quantitative PCR was performed. Total RNA was extracted from each sample and three technical replicates were performed for each of three biological replicates. cDNAs were synthesized using the One-Step gDNA Removal and cDNA Synthesis SuperMix (TransGen Biotech Co., Ltd., Beijing, China) following the kit manual. β-actin was used as a reference gene (accession number-EU585777.1), and it was used to select the cDNA templates on the PCR equipment. Primers (Supplementary Table S2) were designed manually or using the Primer 5 tool3. Individual qRT-PCR reactions were repeated four times; water was used as the negative control. Before gene quantification, the amplification efficiency between the target gene and the reference gene were checked. qRT-PCR reactions were performed on the Applied Bio System 7500 Real-Time PCR System (Applied Biosystems, Foster City, CA, United States) using SYBR Green (TAKARA Bio Inc., Japan) The cycling program consists of initial incubation at 95 °C for 10 min, followed by 40 cycles at 95 °C for 15 s, 60 °C for 45 s, and final step at 95 °C for 15 s and reactions were performed in a final volume of 25 µl. The threshold cycle (CT) was collected from each reaction, and their relative expression of normalized data was calculated by the comparative 2−11 CT method [65].

### 4.7. Statistical Analysis

Data collected from maize physiological and biochemical experiments were analyzed using standard analysis of variance (two-way ANOVA) with factorial arrangement using Statistix 8.1 software. The significance of treatments means at *p* < 0.05 was tested by least significant difference (LSD) test. Alignment of RNA seq. data was done with HISAT2 software [106]. Assembling of transcripts with mapped reads was done by using String Tie [107]. For quantification of expression level of transcripts for each sample we used ASprofile software [108]. Differentially expressed genes were identified by DEseq [109], and functional enrichment analysis was done with R package topGO [110].

## 5. Conclusions

The current study concluded that coculture of *B. bassiana* and *T. asperellum* has the synergistic potential to suppress the immune response of *O. furnacalis* and can be used as sustainable approach to induce plant resistance through activation of defense related enzymes. Binary combination of *B. bassiana* with *T. asperellum* can increase the lethal activity of *T. asperellum*. The seed coating method proved to be most effective to endophytically colonize plants and can help plants to grow well. Through transcriptome analysis, it was speculated that the expression of immune related genes was activated in case of *T. asperellum* inoculation only but in the case of *B. bassiana* and combined treatment the expression was low. Thus, they have the ability to suppress the immune response of *O. furnacalis*. Use of these biopesticides is an eco-friendly and sustainable approach to control insect attack, increase the production of crops and eliminate the use of hazardous chemical pesticides.

## Figures and Tables

**Table 1:** Median lethal concentration (LC_50_) value of *B. bassiana* OFDH1-5 and *T. asperellum* GDFS1009 bioassayed against *O. furnacalis*.

Fungal Strain	LC_50_ (%)	95% FL	Slope ± SE	X^2^ (DF = 13)	*p*-Value
***Beauveria bassiana* OFDH1-5**	5.9 × 10^5^	143015.917–1582815.025	0.436 ± 0.065	7.89	≤0.01
***Trichoderma asperellum* GDFS1009**	8.4 × 10^8^	273575649.988–5512644984.830	0.454 ± 0.079	6.221	≤0.01

**Table 2:** Effect of fungal inoculation on mortality (%) of *O. furnacalis*, number and length of tunnels in maize plants. Each value is the mean of three different replicates ± standard deviation. Treatment details: uninoculated and uninfested control **(C)**; seed coating with *B. bassiana* OFDH1-5 **(T1)**; soil drenching with *B. bassiana* OFDH1-5 **(T2)**; seed coating with *T. asperellum* GDFS1009 **(T3)**; soil drenching with *T. asperellum* GDFS1009 **(T4)**; seed treatment with binary combination of *B. bassiana* OFDH1-5 + *T. asperellum* GDFS1009 **(T5)**; soil drenching with binary combination of *B. bassiana* OFDH1-5 + *T. asperellum* GDFS1009 **(T6)**; infested control (insect control) **(T7)**.

Treatments	Mortality (%)	Tunnel Number	Tunnel Length (cm)
**C**	0 ± 0 ^e^	0 ± 0 ^e^	0 ± 0 ^d^
**T1**	85 ± 1.6 ^a^	1.2 ± 0.79 ^c,d^	0.765 ± 0.75 ^c,d^
**T2**	71.6 ± 4.3 ^b,c^	1.7 ± 0.82 ^b,c^	1.14 ± 0.55 ^b,c,d^
**T3**	53.3 ± 2.2 ^c^	2.3 ± 0.48 ^b^	2.08 ± 0.81 ^b,c^
**T4**	46.67 ± 2.1 ^d^	2.3 ± 0.5 ^b^	2.476 ± 1.06 ^b^
**T5**	91.7 ± 3.7 ^a^	0.6 ± 0.84 ^d,e^	0.49 ± 0.72 ^d^
**T6**	81.6 ± 0.74 ^a,b^	1.1 ± 0.57 ^c,d^	1.208 ± 0.68 ^b,c,d^
**T7**	8.7 ± 2.7 ^e^	4.7 ± 1.16 ^a^	4.64 ± 1.96 ^a^

For each column, different lowercase letters indicate significant differences among the treatments, as measured by one-way ANOVA followed by Tukey’s test (*p* < 0.05).

**Table 3:** Number of differentially expressed genes in *O. furnacalis* in response to fungal infection.

DEG Set	Total	Upregulated	Downregulated
**CONTROL vs. BB**	218	168	50
**CONTROL vs. BB-TH**	38	13	25
**CONTROL vs. TH**	45	17	28

**Table 4:** Summary of immune-related genes identified in *O. furnacalis* transcriptome after entomopathogenic fungal infection.

Gene ID	Function	Expression (Log2)		
		CONTROL vs. BB	CONTROL vs. BB+TH	CONTROL vs. TH
gene-LOC114351388	Signal transduction mechanisms	2.891216	---	--
gene-LOC114359283	serine/threonine-protein kinase	1.801724	--	--
gene-LOC114365584	Signal transduction mechanisms	1.71629	--	--
gene-LOC114363167	Signal transduction mechanisms	1.111284	--	--
gene-LOC114352123	peptidoglycan recognition protein	1.783481	--	--
gene-LOC114359877	chitinase-3	3.204765	--	--
gene-LOC114354452	Signal transduction mechanisms	−1.1389	--	--
gene-LOC114351571	Signal transduction mechanisms	1.841207	--	--
gene-LOC114354308	Defense mechanisms (ABC transporter)	1.421725	--	--
gene-LOC114366083	Signal transduction mechanisms	2.038954	--	--
gene-LOC114350700	Signal transduction mechanisms	2.649408	--	--
gene-LOC114356220	heat shock protein 83	−1.72546	−1.8703	--
gene-LOC114353130	Defense mechanisms (ABC transporter)	2.170025	--	--
gene-LOC114358387	heat shock protein 20.4	−2.57941	−2.9913	--
gene-LOC114353313	UDP-glucosidase	1.953112	--	1.78757
gene-LOC114362284	Signal transduction mechanisms	1.597204	--	--
gene-LOC114359576	serine/threonine-protein kinase	1.717456	--	--
gene-LOC114362676	serine/threonine-protein kinase	1.821126	--	--
gene-LOC114365247	Signal transduction mechanisms	1.355035	--	--
gene-LOC114364856	Signal transduction mechanisms	1.350309	--	--
gene-LOC114357915	Signal transduction mechanisms	1.705776	--	--
gene-LOC114357147	Signal transduction mechanisms	1.634834	--	--
gene-LOC114362187	Signal transduction mechanisms	1.942793	--	--
gene-LOC114351255	serine/threonine-protein phosphatase	1.745418	--	--
gene-LOC114355543	Signal transduction mechanisms	1.109096	--	--
gene-LOC114366552	Insect cuticle protein	−1.75469	--	--
gene-LOC114354721	heat shock protein 24.3	−2.38213	−3.15665	--
gene-LOC114351436	serine proteinase inhibitor	2.446235	--	--
gene-LOC114352122	peptidoglycan-recognition protein-S	2.992085	--	--
gene-LOC114354585	peptidoglycan-recognition protein-S	1.810414	--	--
gene-LOC114356221	Heat shock protein 70	−2.19843	−2.84068	−1.96491
gene-LOC114354346	Signal transduction mechanisms	1.478152	--	--
gene-LOC114351799	Signal transduction mechanisms	1.927021	--	--
gene-LOC114351742	Signal transduction mechanisms	1.262353	--	--
gene-LOC114366353	Insect cuticle protein	−2.04885	−1.34725	2.27946
gene-LOC114362562	Cadherin	1.536785	0.9054481	0.7932067
gene-LOC114366367	Insect cuticle protein	−1.66763	−1.26854	1.5968267
gene-LOC114354308	ACB transporter	1.421725	1.1354	1.11238
gene-LOC114350074	UDP-glucuronosyltransferase	−1.41575	−0.9498	−1.30987
gene-LOC114359337	Signal transduction mechanisms	--	2.726825	--
gene-LOC114356222	Protein lethal essential for life (HSP-20)	--	−2.80833	−2.34904
gene-LOC114363773	Heat shock protein 68	−4.13625	−3.49075	−3.89546
gene-LOC114353087	Cytochrome P450	−0.99134	−1.6274	−0.23413
gene-LOC114354720	Protein lethal (2) essential for life	--	−2.25955	−1.99824
gene-LOC114352113	Peptidoglycan-recognition protein-B		−2.05588	−2.1622
gene-LOC114353764	Protein lethal essential for life (HSP- 20)	--	−3.00427	−1.69466
gene-LOC114358386	Protein lethal essential for life (HSP−20)	--	−2.45793	--
gene-LOC114357364	Defense mechanisms (ABC transporter)	--	3.555454	--

## Supplementary Materials

The following are available online at https://www.mdpi.com/1422-0067/21/21/8215/s1.
