# Supplementary material for: Synergistic Effect of Beauveria bassiana and Trichoderma asperellum to Induce Maize (Zea mays L.) Defense against the Asian Corn Borer, Ostrinia furnacalis (Lepidoptera, Crambidae) and Larval Immune Response
Source: Int J Mol Sci. 2020 Nov 3;21(21):8215. doi: 10.3390/ijms21218215 (PMC7663379; doi:10.3390/ijms21218215)
Supplement: Supplementary file 1 [file ijms-21-08215-s001.pdf]

Supplementary Data:

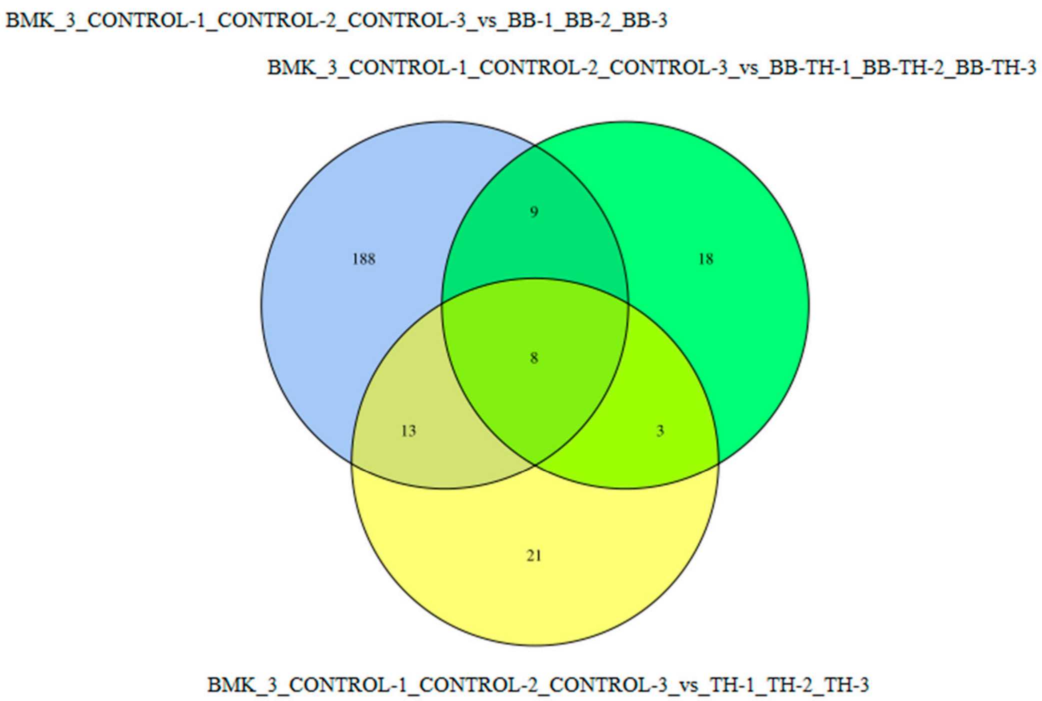

Figure S1. *Venn diagram of differentially expressed genes.*

Table S1. *Summary of reads from O. furnacalis transcriptome after fungal inoculation.*

Samples	Clean reads	Total mapped	GC (%)	Q30 (%)
**CONTROL-1**	47,663,380	41,500,043 (87.07%)	49.05%	95.98%
**CONTROL-2**	56,855,716	49,259,914 (86.64%)	48.00%	95.79%
**CONTROL-3**	55,350,190	47,655,803 (86.10%)	48.41%	95.77%
**BB-1**	49,557,902	42,803,307 (86.37%)	48.91%	95.90%
**BB-2**	54,617,758	47,383,866 (86.76%)	48.43%	96.14%
**BB-3**	49,090,076	41,044,436 (83.61%)	48.59%	95.90%
**BB-TH-1**	44,013,916	37,579,113 (85.38%)	48.99%	95.09%
**BB-TH-2**	44,801,702	38,775,359 (86.55%)	48.97%	95.48%
**BB-TH-3**	49,374,034	42,763,282 (86.61%)	49.46%	95.17%
**TH-1**	63,186,052	53,128,376 (84.08%)	48.70%	95.79%
**TH-2**	42,901,514	36,479,674 (85.03%)	48.87%	95.57%
**TH-3**	48,067,818	39,696,165 (82.58%)	48.65%	95.63%

Table S2. *Primers used for RT-qPCR*

Gene name	Gene I.D	Direction	Sequance (5'-3')
**PRPS**	gene-LOC114352122	Forward	TGCAGAGGTACCACAACAGC
		Reverse	GCTGACTCCGTTGGCTCTAC
**PRPB**	gene-LOC114352113	Forward	GAAAAATGGCCCGTCTTACA
		Reverse	CGTCACGAACGGGAAGTTAT
**Larval/pupal rigid cuticle protein**	gene-LOC114366353	Forward	ATGGCAGCTAAGTTCGTCGT
		Reverse	CCGTACGAGAAGCTGGTGTAG
**Larval/pupal rigid cuticle protein 2**	gene-LOC114366367	Forward	GGGATCAATCATTCGCAGTC
		Reverse	ACCAGGAGAGCAACAACGAC
**Cytochrome P450**	gene-LOC114353087	Forward	AGAGCGACTGGTGAAGGAAA
		Reverse	CCTCATCAGCTCTGACACCA
**ABC transporter 1**	gene-LOC114354308	Forward	AACCACATGACATTGGCTCA
		Reverse	ACGCGATCGAGCTTCAGTAT
**ABC transporter 2**	gene-LOC114353130	Forward	TACCCCCAGAAACTCCAGTG
		Reverse	TTTGGCGGTTTCTCGTATTC
**Cadherin**	gene-LOC114362562	Forward	CAACCAACCAGCATTTTGTG
		Reverse	TCTTTTGACAGCTCGGGTCT
**UDP-glucuronosyltransferase**	gene-LOC114350074	Forward	GAGTGCCCTTCGAGACTCTG
		Reverse	AATCGAGGCCGAGTCCTTAT
**HSP-70**	gene-LOC114356221	Forward	AAGAACCAGGTCGCTCTCAA
		Reverse	AAGGGCCAGTGTTTCATGTC
**HSP-68**	gene-LOC114363773	Forward	ACATGAAACACTGGCCCTTC
		Reverse	TACGCCTCTGCAGTTTCCTT
